# Imaging Memory T-Cells Stratifies Response to Adjuvant Metformin Combined with αPD-1 Therapy

**DOI:** 10.3390/ijms232112892

**Published:** 2022-10-25

**Authors:** Julian L. Goggi, Siddesh V. Hartimath, Shivashankar Khanapur, Boominathan Ramasamy, Zan Feng Chin, Peter Cheng, Hui Xian Chin, You Yi Hwang, Edward G. Robins

**Affiliations:** 1 Institute of Bioengineering and Bioimaging (IBB), Agency for Science, Technology and Research (A*STAR), 11 Biopolis Way, #01-02 Helios, Singapore 138667, Singapore; 2 Singapore Immunology Network (SIgN), Agency for Science, Technology and Research (A*STAR), 8A Biomedical Grove, Immunos, Singapore 138648, Singapore; 3 Clinical Imaging Research Centre (CIRC), Yong Loo Lin School of Medicine, National University of Singapore, 14 Medical Drive, #B1-01, Singapore 117599, Singapore

**Keywords:** immune checkpoint inhibitors (ICI), positron emission tomography (PET), potassium channels, metformin

## Abstract

The low response rates associated with immune checkpoint inhibitor (ICI) use has led to a surge in research investigating adjuvant combination strategies in an attempt to enhance efficacy. Repurposing existing drugs as adjuvants accelerates the pace of cancer immune therapy research; however, many combinations exacerbate the immunogenic response elicited by ICIs and can lead to adverse immune-related events. Metformin, a widely used type 2 diabetes drug is an ideal candidate to repurpose as it has a good safety profile and studies suggest that metformin can modulate the tumour microenvironment, promoting a favourable environment for T cell activation but has no direct action on T cell activation on its own. In the current study we used PET imaging with [^18^F]AlF-NOTA-KCNA3P, a radiopharmaceutical specifically targeting K_V_1.3 the potassium channel over-expressed on active effector memory T-cells, to determine whether combining PD1 with metformin leads to an enhanced immunological memory response in a preclinical colorectal cancer model. Flow cytometry was used to assess which immune cell populations infiltrate the tumours in response to the treatment combination. Imaging with [^18^F]AlF-NOTA-KCNA3P demonstrated that adjuvant metformin significantly improved anti-PD1 efficacy and led to a robust anti-tumour immunological memory response in a syngeneic colon cancer model through changes in tumour infiltrating effector memory T-cells.

## 1. Introduction

Immune checkpoint inhibitors (ICIs) have achieved great success in the field of immuno-oncology, but low response rates have prompted research to investigate combination strategies in an attempt to enhance efficacy [1,2]. Repurposing existing drugs as adjuvants for ICI treatment is a promising course of action and accelerates the pace of cancer immune therapy research. Clinical trials to assess the use of viruses, tumour vaccines, chemotherapy and molecular targeted drugs are underway to determine which adjuvants are suitable to improve response rates when combined with ICIs [3]. Most adjuvants being tested have demonstrated independent immune stimulatory effects and either exhibit their own immune related side effects or have the potential to exacerbate those commonly associated with ICI therapy [4,5]. Metformin, however, is an ideal candidate to repurpose as it is one of the most widely used drugs for patients with type 2 diabetes, has been linked with a preventative role in cancer formation, and has an excellent safety profile [6]. Metformin regulates the adenosine monophosphate-activated protein kinase (AMPK) and liver kinase B1 (LKB1) pathways, which inhibit the mammalian target of rapamycin (mTOR). This results in the inhibition of protein synthesis, gluconeogenesis, and insulin production [7,8] all of which can help regulate tumour growth. Recent studies have suggested that metformin may also enhance tumouricidal immune responses when paired with ICIs by reducing tumour hypoxia, a barrier to successful tumour immune-responses, improving T-cell proliferation and effector function [9]. One recent clinical study attributes metformin with overcoming resistance to nivolumab in a patient with lung cancer by reducing tumour hypoxia, although the study size was small [10], while another showed improved responses in diabetic melanoma, renal cell carcinoma and lung cancer patients [11]. Other studies have shown limited effects of combination with metformin [6,12]; however, most studies showed that the combined use of nivolumab with metformin was safe and did not increase the risk of adverse events [13]. Determining whether drugs with relatively mild effects on the tumour microenvironment can improve ICI response is especially difficult as the current methods for assessing treatment efficacy are insensitive and mainly geared towards measuring changes in tumour volumes [14]. K_V_1.3 is overexpressed in active effector memory T-cells (T_EM_) [15], and is involved in the development of a robust anti-tumour immunological memory response. Head and neck cancer patients that responded to ICI therapy with PD1 inhibitors had TILs with high expression of Kv1.3 [16] and significant increases in T_EM_ cells in responsive tumours versus non-responsive tumours [17]. Recently we demonstrated that [^18^F]AlF-NOTA-KCNA3P, a radiopharmaceutical specifically targeting K_V_1.3, is able to reproducibly identify lasting therapy response to ICIs [18]. In the current study we demonstrate that [^18^F]AlF-NOTA-KCNA3P is sensitive enough to measure an enhanced immunological memory response induced by combining αPD1 with metformin.

## 2. Results

### 2.1. Model Development and Evaluation of Treatment Efficacy

**Figure 1:**
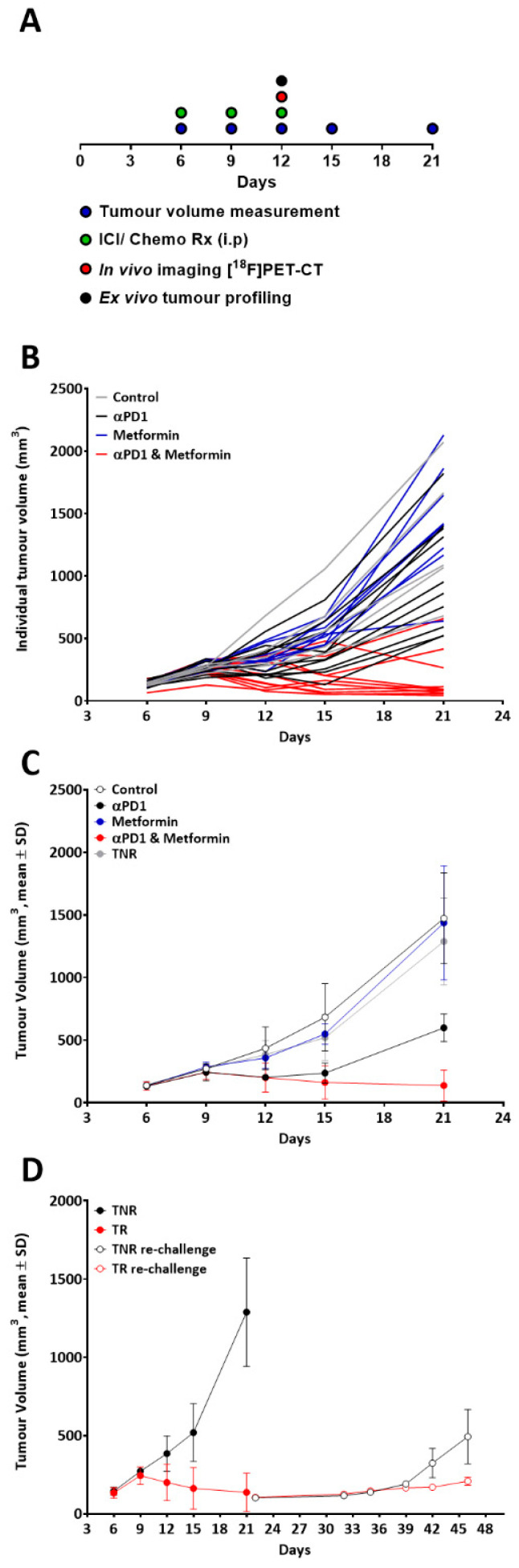
(**A**) Schematic showing the treatment, tumour volume assessment and imaging regimen. (**B**) Individual tumour volumes for each animals highlighting the variability in response. (**C**) Tumour volumes in each treatment cohort after therapy response stratification. (**D**) Average tumour volume after tumour re-challenge. Data are represented as mean ± S.D. (TR, responding tumours; TNR, non-responding tumours).

A schematic representing the treatment regimen, tumour volume assessment and imaging for the animals is shown in Figure 1A. Overall, tumour growth showed normal distribution with each treatment cohort exhibiting different response rates and magnitudes (Figure 1B, Shapiro–Wilk *p* = 0.881). The greatest response rate and tumour shrinkage was observed in the combined αPD1 plus Metformin treatment group, significantly greater than observed in the αPD1 monotherapy treated group, while metformin alone had no significant effect on tumour growth (Figure 1C and Supplementary Table S1). The criteria for separation of tumours into treated responders (TR) or treated non-responders (TNR) has been described previously [19] and is dependent on the change in tumour volume between the first tumour volume measurement on day 6 and the final tumour volume measurement on day 21 for each individual animal, using the control treated group tumours as a reference point for TNRs. In the current study, TR animals displayed tumour volumes ≤ 740 mm^3^ on day 21 (>2 SD lower than the mean control group value on day 21).

Importantly, TR animals displaying high tumour retention of [^18^F]AlF-NOTA-KCNA3P (on day 12) showed little tumour growth after re-challenge with tumour cells; however, TNR animals displaying low tumour retention of [^18^F]AlF-NOTA-KCNA3P (on day 12) showed significantly greater tumour growth after re-challenge (Figure 1D).

### 2.2. Tumour Retention of [^18^F]AlF-NOTA-KCNA3P Assessed by PET Imaging

**Figure 2:**
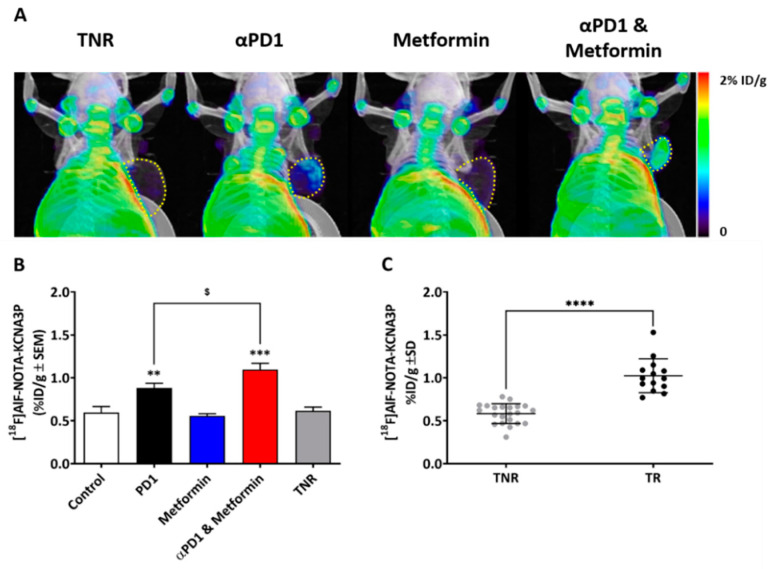
(**A**) Representative MIP images indicating tumour retention of [^18^F]AlF-NOTA-KCNA3P across the treatment cohorts. Tumour borders are shown as yellow dotted lines. (**B**) Bar graph indicating tumour retention of [^18^F]AlF-NOTA-KCNA3P in each treatment cohort (Control, αPD1, metformin, combined αPD1 + metformin and TNRs; *n* = 5–10 mice/group; ** *p* < 0.01, *** *p* < 0.001 compared to TNR, ^$^ *p* < 0.05 compared to αPD1; data shown as the mean %ID/g ± S.E.M.). (**C**) Retention of [^18^F]AlF-NOTA-KCNA3P in individual tumours from TR and TNRs (**** *p* < 0.0001).

Tumour retention of [^18^F]AlF-NOTA-KCNA3P varied across the different treatment cohorts studied (Figure 2A and Table 1). Tumour retention of [^18^F]AlF-NOTA-KCNA3P and tumour growth inhibition were well correlated (Pearson r = 0.668, *** *p* < 0.01, *n* = 50). The control cohort, the metformin cohort and the TNR showed little [^18^F]AlF-NOTA-KCNA3P tumour retention, whereas the tumours responsive to αPD1 had significantly greater retention (** *p* < 0.01 compared to TNR). The αPD1 + metformin combination responders, however, showed even greater retention of [^18^F]AlF-NOTA-KCNA3P when compared to the TNRs (*** *p* < 0.001) and significantly increased retention compared to the αPD1 responsive group ($ < 0.05, Figure 2).

### 2.3. Tumour Retention of [^18^F]AlF-NOTA-KCNA3P Is Associated with Tumour Infiltration of K_V_1.3 Expressing T_EM_ Cells

**Figure 3:**
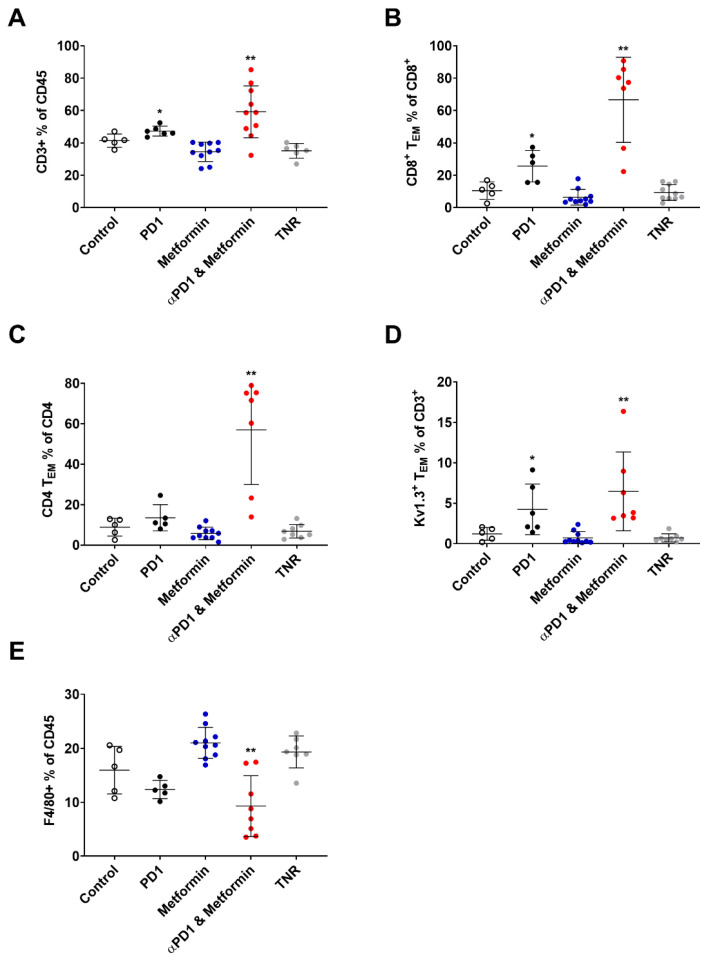
Tumour infiltrating immune cell populations determined using FACS across each treatment cohort (control, αPD1, metformin, combined αPD1 + metformin and TNRs). Data shown as (**A**) CD3^+^ cells as a % of total CD45^+^ cells, (**B**) CD8^+^ T_EM_ cells as a % of total CD8^+^ cells, (**C**) CD4^+^ T_EM_ cells as a % of total CD4^+^ cells, (**D**) K_V_1.3^+^ T_EM_ cells as a % of total CD3^+^ cells, and (**E**) F4/80^+^ cells as a % of total CD45^+^ cells. Data indicated are individual values with mean ± S.D. representative of *n* = 5–10 mice/cohort. * *p* < 0.05; ** *p* < 0.01 compared to TNR.

Immunophenotypic populations associated with tumours designated as treated responders (TR) were compared to tumours designated as treated non-responders (TNR) assessed using flow cytometry (Figure 3). Changes in the tumour infiltrating immunophenotype were clear in tumours responding to αPD1 or combined αPD1 + metformin with the greatest changes associated with CD8^+^ T_EM_ cells and CD4^+^ T_EM_ cells (Figure 3B,C and Table 2).

## 3. Materials and Methods

### 3.1. [^18^F]AlF-NOTA-KCNA3P Radiochemistry

The precursor NOTA-KCNA3P peptide was custom synthesized by the Chinese Peptide Company (CPC) and radiolabeling was performed as previously described [18]. [^18^F]AlF-NOTA-KCNA3P was isolated with a non-decay corrected radiochemical yield of 22.0 ± 6.4% within 50 min from delivery of aqueous [^18^F]fluoride. The radiochemical purity was greater than 99% and the molar activity was 32.5 ± 11.2 GBq/µmol at the end of synthesis (*n* = 4, Supplementary Materials Section S1.3).

### 3.2. Animal Procedures

All animal procedures adhered to the Singapore Institutional Animal Care and Use Committee regulations (IACUC No. 211649). The tumour implantation procedure was carried out as previously reported [19]. Mice were purchased from InVivos Singapore (BALB/c, 5–7 weeks old) and CT26 cells were implanted into the right shoulder (2 × 10^5^ cells per animal). The mice were dosed IP on days 6, 9 and 12 following tumour implantation with either 5 mg/kg rat IgG2a isotype control (α-trinitrophenol mAb) or 10 mg/kg rat IgG2a anti-mouse PD-1 (αPD1 mAb RMP1-14, Bio-X-Cell, New Hampshie USA). Metformin was dosed IP on days 6, 8, 10 and 12 following tumour implantation (50.0 mg/kg, Sigma-Aldrich, Singapore, Singapore). Tumour volumes were measured longitudinally using calipers, and the tumour response was determined by measuring tumour growth inhibition as described in the Supplementary Materials Section S1.4.

On day 22 post implantation, animals with tumours responsive to therapy (TR) and concomitant high [^18^F]AlF-NOTA-KCNA3P tumour retention (>0.8%ID/g), and animals with tumours that were non-responsive to therapy (TNR) and low [^18^F]AlF-NOTA-KCNA3P tumour retention (<0.5%ID/g), were reimplanted with CT26 tumour cells in the contralateral left shoulder as described above. Tumour growth was assessed for a further 25 days.

### 3.3. PET-CT Imaging

A Siemens Inveon PET-CT was used to image the animals at 12 days after tumour implantation, as described previously [18]. [^18^F]AlF-NOTA-KCNA3P (~10 MBq) was injected via the lateral tail vein and tissue retention assessed using static PET imaging at 60 min post injection. Amide software (version 10.3 Sourceforge, Stanford, CA, USA) was used to analyse the static acquisitions and delineate volumes of interest to determine radioactivity retention in tissues. Data are expressed as % of the injected dose per gram (%ID/g).

### 3.4. Flow Cytometry

Flow cytometry was used to assess the tumour-infiltrating immune cells as described in detail previously [19]. The tumours were excised and processed into a single cell suspension, assessed for viability with Trypan Blue (Sigma-Aldrich), and stained for a wide range of immune cell markers, as detailed in the Supplementary Materials Section S1.5. Flow cytometry was performed on a BD FACSymphony. Data were recompensated and analysed using FlowJo V10.7.1 software (FlowJo LLC, Ashland, OR, USA). Dimension reduction analysis has also been detailed previously. t-Distributed Stochastic Neighbor Embedding (t-SNE) was used for unbiased dimension reduction and Rphenograph was used for clustering using the default parameters with the cytofkit package in RStudio [18,20] (https://github.com/JinmiaoChenLab/cytofkit, accessed on 2 May 2022). One thousand cells from each fcs file were used for analysis, using the following markers: K_V_1.3 (K_V_1.3 potassium channel marker), CCR7 (memory T cell marker), CD3 (pan-T cell marker), CD4 (helper T cell marker), CD8 (cytotoxic T cell marker), CD11b (myeloid marker), CD11c (dendritic cell marker), CD206, F4/80, I-A/I-E (MHC class II marker), Ly6C (macrophage marker), Ly6G (neutrophil marker), Nkp46 (pan-NK cell marker) and Siglec-F (eosinophil marker).

### 3.5. Statistical Analysis

Kruskal Wallis 1-way ANOVA with Dunn’s post-test for multiple comparisons were used for statistical analysis of the non-parametric data sets (GraphPad Prism version 8.3.4, GraphPad Software, San Diego, CA, USA). *p* < 0.05 was considered statistically significant. Data are expressed as mean ± S.D. unless otherwise indicated.

## 4. Discussion

[^18^F]AlF-NOTA-KCNA3P reproducibly measured anti-tumour immunological memory T cell responses with increased retention, evident in tumours responding to PD-1 inhibition and significantly augmented after adjuvant metformin therapy. However, metformin had no effect on [^18^F]AlF-NOTA-KCNA3P retention alone (Figure 2). Metformin has been shown to play a preventative role in many cancer types, including colorectal cancer, reducing incidence and mortality [21]; however, this is not mediated through direct modulation of tumour infiltrating T cells. This preventative role is mediated in part by metformin’s ability to regulate glucose, modulating its availability to both the tumour and cells in the tumour microenvironment [22]. Metformin also reduces tumour oxygen consumption and hypoxia. These changes to the tumour microenvironment encourage T-cells to proliferate in a regulated way once stimulated by a blockade of PD-1 [10]. The blockade of PD-1 can have a profound effect on the tumour microenvironment, activating tumour-associated T-cells and reinvigorating exhausted CD8^+^ T cells, which can lead to tumor regression [17]. However, the tumour microenvironment contains many types of immune cells, including immune suppressor cells such as tumour-associated macrophages (TAMs), myeloid derived suppressor cells (MDSCs), and regulatory T-cells (Treg cells), which can interfere with the efficacy of PD-1 blockade [10]. When used as an adjunct, metformin’s ability to modulate the tumour microenvironment can significantly improve the magnitude and durability of tumour-infiltrating T-cells in response to the PD-1 blockade [9]; however, metformin has little effect on the quantity or type of tumour infiltrating immune cells when delivered alone (Figure 3). Figure 3A shows a significant increase in CD3^+^ T-cells and Figure 3E, and a concomitant decrease in immune suppressive F480^+^ cells after combined PD1 and metformin treatment. This reduction in immune suppression mediated by metformin promotes T-cell differentiation in response to antigen stimulation. After antigen stimulation, naïve T-cells differentiate into effector T-cells or memory T-cells and undergo metabolic reprogramming. Naïve T-cells use oxidative phosphorylation as an energy source, whereas effector T-cells rely on aerobic glycolysis. This metabolic reprogramming is initiated by the phosphatidylinositol-3- kinase–protein kinase B–mammalian target of the rapamycin (PI3K–AKT–mTOR) pathway during T-cell activation, and can lead to T-cell exhaustion [7,23,24]. Exhausted T-cells lack tumouricidal ability and hamper the effective functioning of ICIs [24]. Metformin blocks mTOR signalling, moderating effector T-cell expansion, which reduces exhaustion, restoring oxidative phosphorylation and promoting differentiation to memory T-cells [25]. Figure 3B,C clearly shows significantly increased tumour-associated CD4^+^ T_EM_ and CD8^+^ T_EM_ cells after combined αPD1 and metformin treatment, whereas αPD1 monotherapy only leads to a moderate increase in CD8^+^ T_EM_ cell infiltration, and metformin monotherapy causes no significant change in comparison to the non-responding tumours. This increase in tumour-infiltrating T_EM_ cells has the potential to inhibit further tumour growth, as shown by the CT26 tumour re-challenge (Figure 1D), highlighting the importance of non-invasive measurement of tumour-infiltrating T_EM_ cells. The presence of high numbers of infiltrating T_EM_ cells has been shown to correlate with a reduction in metastatic invasion and improved survival [26], suggesting that αPD1-combinations that increase T_EM_ cells have the potential to abrogate tumour metastasis in colon cancer patients.

Whether [^18^F]AlF-NOTA-KCNA3P may prove effective clinically remains to be assessed. Tumour uptake of [^18^F]AlF-NOTA-KCNA3P is intratumoural, but may be affected by tissue necrosis or changes in vascularity, as K_V_1.3 is expressed in endothelial cells. Interpretation of tissue retention can be complicated by background uptake. K_V_1.3 has been shown to be expressed by some cancers [27,28,29,30,31,32,33], immune cells including B lymphocytes and macrophages [34], and high uptake has been observed at the bone epiphyseal endplates [35], potentially obfuscating uptake in some tissues.

## 5. Conclusions

Imaging with [^18^F]AlF-NOTA-KCNA3P provides a non-invasive way to measure tumour infiltrating memory T-cells associated with metformin’s ability to enhance αPD1 response. Tumours exhibiting higher [^18^F]AlF-NOTA-KCNA3P retention demonstrated an on-going resistance to tumour cell re-challenge, suggestive of a durable anti-tumour memory response. The data indicate that [^18^F]AlF-NOTA-KCNA3P may be able to distinguish which drugs can be repurposed successfully as adjuvants to enhance the efficacy and durability of PD1 checkpoint inhibition clinically.

## Figures and Tables

**Table 1:** Table displaying [^18^F]AlF-NOTA-KCNA3P tumour retention in each treatment cohort (control, αPD1, metformin, combined αPD1 + metformin and TNRs).

Treatment Group	[^18^F]AlF-NOTA-KCNA3P Tumour Retention (%ID/g ± SD)
Control	0.596 ± 0.173
Treatment Responders (TR) αPD1	0.883 ± 0.120 **
Metformin	0.557 ± 0.082
PD1 + Metformin	1.098 ± 0.203 *** ^$^
Treated Non-Responders (TNR)	0.617 ± 0.105

Data are shown as the mean %ID/g ± S.D; *n* = 5–10 mice/group; ** *p* < 0.01, *** *p* < 0.001 comparing to TNR, ^$^ *p* < 0.05 comparing to αPD1 alone.

**Table 2:** Percentages of immune cell populations across each treatment cohort (control, αPD1, metformin, combined αPD1 + metformin and TNRs).

	Immune Cell Subpopulations Associated with CT26 Tumours				
Treatment Cohort	CD3^+^ % of CD45^+^	CD8^+^ T_EM_ % of CD8^+^	CD4^+^ T_EM_ % of CD4^+^	K_V_1.3^+^ T_EM_ % of CD3^+^	F4/80^+^ % of CD45^+^
Control	41.41 ± 4.08	10.53 ± 5.35	8.87 ± 4.39	1.21 ± 0.79	15.98 ± 4.41
TR					
αPD1	47.28 ± 3.04 *	25.64 ± 9.78 *	13.51 ± 6.49	4.24 ± 3.13 *	12.39 ± 1.70
Metformin	34.48 ± 5.97	6.43 ± 4.84	5.82 ± 3.08	0.71 ± 0.57	21.03 ± 2.85
αPD1 + Metformin	59.23 ± 16.02 **	66.65 ± 26.31 **	59.96 ± 26.97 **	6.47 ± 4.86 **	9.31 ± 5.64 **
TNR	35.13 ± 4.57	9.35 ± 4.84	6.86 ± 3.40	0.72 ± 0.51	19.33 ± 2.95

Data are shown as the mean % of cells ± S.D; *n* = 5–10 mice/group; * *p* < 0.05, ** *p* < 0.01 compared to TNR.

## Data Availability

The data presented in this study are available on request from the corresponding author.

## Supplementary Materials

The following supporting information can be downloaded at https://www.mdpi.com/article/10.3390/ijms232112892/s1. Reference [36] is cited in the supplementary materials.
