# Supplementary material for: Imaging Memory T-Cells Stratifies Response to Adjuvant Metformin Combined with αPD-1 Therapy
Source: Int J Mol Sci. 2022 Oct 25;23(21):12892. doi: 10.3390/ijms232112892 (PMC9654631; doi:10.3390/ijms232112892)
Supplement: Supplementary file 1 [file ijms-23-12892-s001.zip › ijms-1967793-supplementary.pdf]

International Journal of *Molecular Sciences*

MDPI

Article

Imaging Memory T-Cells Stratifies Response to Adjuvant Metformin Combined with PD-1 Therapy

Julian L. Goggi [1,*], Siddesh V. Hartimath [1], Shivashankar Khanapur [1], Boominathan Ramasamy [1], Zan Feng Chin [1], Peter Cheng [1], Hui Xian Chin [2], You Yi Hwang [2] and Edward G. Robins [1,3]

1 Institute of Bioengineering and Bioimaging (IBB), Agency for Science, Technology and Research (A*STAR), 11 Biopolis Way, #01-02 Helios, Singapore 138667, Singapore.
2 Singapore Immunology Network (SIgN), Agency for Science, Technology and Research (A*STAR), 8A Biomedical Grove, Immunos, Singapore 138648, Singapore University of Singapore, 14 Medical Drive, #B1-01, 117599, Singapore.
3 Clinical Imaging Research Centre (CIRC), Yong Loo Lin School of Medicine, National University of Singapore, 14 Medical Drive, #B1-01, Singapore 117599, Singapore
* Correspondence: julian_goggi@ibb.a-star.edu.sg; Tel.: +65-6824-7093

Citation: Goggi, J.L.; Hartimath, S.V.; Khanapur, S.; Ramasamy, B.; Chin, Z.F.; Cheng, P.; Chin, H.X.; Hwang, Y.Y.; Robins, E.G. Imaging Memory T-Cells Stratifies |Response to Adjuvant Metformin Combined with PD-1 Therapy. *Int. J. Mol. Sci.* **2022**, *23*, 12892. https://doi.org/10.3390/ijms232112892

Academic Editors: Benito Antonio Yard and Jan Leipe

Received: 28 September 2022
Accepted: 14 October 2022
Published: 25 October 2022

Publisher's Note: MDPI stays neutral with regard to jurisdictional claims in published maps and institutional affiliations.

Copyright: © 2022 by the authors. Submitted for possible open access publication under the terms and conditions of the Creative Commons Attribution (CC BY) license (https://creativecommons.org/licenses/by/4.0/).

S1.1 Supplementary General information

Aluminium chloride (≥ 99.999%) and sodium fluoride were procured from Sigma-Aldrich Pte Ltd, Singapore. Glacial acetic acid and Saline solution (0.9% w/v) were purchased from JT Baker and Braun Medical Industries, respectively. All other reagents were procured from Merck, VWR chemicals and Fisher Scientific. All commercially obtained reactants and reagents were used as such without any further purification. [18F]AlF-NOTA-KCNA3P synthesis was carried out in a closed Thermo Scientific™ conical reacti-vial™ (1 ml). Sep-Pak® light (46 mg) accell™ plus QMA carbonate (Part No.: 186004540), Oasis HLB plus light cartridge (Part No.: WT186005125) and Sep-Pak C18 plus short cartridges (Part No.: WAT020515) were purchased from Waters Pacific Pte Ltd, Singapore.

No-carrier-added (nca) aqueous [18F]fluoride ion was produced by the irradiation of 18O-enriched water via the [18O(p,n)18F] nuclear reaction using a GE PETtrace 860 cyclotron. Radiochemical purification was performed on a Knaur semi-preparative radio-HPLC system comprising of two Knaur Smartline 1050 pumps, Manual injection valve (6-port/3-channel), SmartMix 100 solvent mixer, Smartline UV-Detector 2520 and Flow-Count radio-HPLC NaI detection system. Quality control analytical radio-HPLC was performed on an UFLC Shimadzu radio-HPLC system equipped with dual wavelength UV detector and a NaI/PMT-radiodetector (Flow-Ram, LabLogic). Radioactivity measurements were made with a CRC-55tPET dose calibrator (Capintec, USA).

LC-MS 2020 (Shimadzu Asia Pacific Pte Ltd) was used for identification of the labelled peptide. The mass spectrometer was operated in electrospray positive ionization mode. The mass spectrometer settings were optimized as follows: interface voltage, 4.5 kV; nebulizer gas flow, 1.5 L / min; drying gas flow, 15 L / min; desolvation line (DL) temperature, 250° C; heat block temperature, 250° C. Other mass spectrometer parameters were tuned automatically. Mass spectral data analysis was done manually for identification of labelled peptide.

S1.2 Supplementary Peptide Synthesis Details

The NOTA-KCNA3P precursor peptide, NOTA-RTCESASH-KFEGPCLRDSNCANVCKTEGFH-GGKCKGLRRRCFCTKHC (Cys1 & Cys8, Cys2 & Cys5, Cys3 & Cys6, Cys4 & Cys7 bridges) was custom synthesized by Chinese Peptide Company (CPC) with >95% purity.

S1.3 Supplementary Details on the Radiosynthesis of [^{18}F]AlF-NOTA-KCNA3P

Aqueous nca [^{18}F]fluoride (typically 10 GBq in 2.4 ml) was trapped on a Sep-Pak® light (46 mg) Accell™ plus QMA carbonate cartridge (preconditioned with 10 ml deionised water) and washed with a further 5 ml of water. The trapped [^{18}F]fluoride anion was then eluted with 0.9% w/v saline (0.2 ml) into a 1.5 ml Eppendorf tube. To the [^{18}F]fluoride solution was added a 24 µl aliquot of a 2 mM $AlCl_3$ stock solution prepared in 0.1 M sodium acetate buffer and pH adjusted to pH 4 using glacial acetic acid. This combined reaction mixture was transferred into a 1 ml reactor vial containing NOTA-KCNA3P (0.2-0.25 mg) followed by an equal volume of ethanol (0.2 ml). The reaction vial was then sealed and heated at 100° C for 15 min without stirring. After cooling to room temperature, the crude reaction mixture was diluted with water (3 ml) and subjected to purification by semi-preparative radio-HPLC (Aeris™ 5µm PEPTIDE XB-C18, 100 Å, 250 x 21.2 mm; 10 ml / min, λ = 254 nm). Gradient elution was carried out using a mixture of 0.1 % aqueous trifluoroacetic acid (solvent A) and 0.1 % trifluoroacetic acid in acetonitrile (solvent B). The following gradient elution profile was used: 0.01 - 0.20 min 10 % B, 0.20 - 8.00 min 70 % B, 8.00 - 10.00 min 70 % B, 11.00 - 15.00 min 10 % B. The retention time of [^{18}F]AlF-NOTA-KCNA3P was between 8.2 - 8.3 min. The collected HPLC pure fraction was then trapped on a pre-conditioned Sep-Pak Plus C18 cartridge (Preconditioning was done using 5 ml ethanol followed by 10 ml deionised water). The cartridge was then washed with 10 ml of water. [^{18}F]AlF-NOTA-KCNA3P was eluted with 70% ethanol in saline (0.5 ml), and diluted with 0.9% w/v saline to a final concentration of 10% ethanol in saline. The radiochemical purity of [^{18}F]AlF-NOTA-KCNA3P was assessed by analytical radio-HPLC (Aeris™ 5µm PEPTIDE XB-C18, 100 Å, 250 x 4.6 mm; 1 ml / min, λ = 220 nm). Gradient elution was carried out using a mixture of 0.1 % aqueous trifluoroacetic acid (solvent A) and 0.1 % trifluoroacetic acid in acetonitrile (solvent B). The following gradient elution profile was used: 0.01 - 0.20 min 10 % B, 0.20 – 10.00 min 70 % B, 10.50 - 16.00 min 10 % B. The retention time of [^{18}F]AlF-NOTA-KCNA3P was 7.5-7.6 min. [^{18}F]AlF-NOTA-KCNA3P was isolated with a non-decay corrected radiochemical yield of 22.0 ± 6.4 % within 50 min (n = 4) from aqueous [^{18}F]fluoride. The radiochemical purity was greater than 99% and molar activity was 32.5 ± 11.2 GBq / µmol at the end of the synthesis.

S1.4 Supplementary Animal Procedures Details

All animal procedures were carried out following the guidelines of the Institutional Animal Care and Use Committee Singapore (IACUC No. 211649). BALB/c mice aged 5-7 weeks were purchased from InVivos (Singapore). Mice were housed in specific-pathogen-free (SPF) environment during the experiments, at room temperature with a 12-hour light-dark cycle and had free access to food and water. The murine colon tu-

mour cell line CT26 was acquired from ATCC and cultured in RPMI supplemented with 10% foetal bovine serum, 100 U/mL penicillin and 100 µg/mL streptomycin, at 37°C in a humidified atmosphere at 5% CO2. CT26 cells (2 x 105) were prepared in a 1:1 (v:v) ratio in Matrigel (Sigma) and injected subcutaneously into the right shoulder of Balb/c mice. In vivo subcutaneous tumours were measured by callipers on days 6, 9, 12, 15, 19 and 21 after tumour inoculation (Supplementary Table S1). Tumour volume was then calculated using the modified ellipsoid formula 1/2(Length × Width2) [1]. In order to accurately assess tumour response to therapy tumour growth inhibition (%TGI) was determined using the formula %TGI = (Vc-Vt)/(Vc-Vo) × 100, where Vc and Vt are the mean tumour volumes of control and treated groups on day 21 and Vo is the tumour volume at the start of the study.

S1.5 Supplementary Flow Cytometry Details

Tumours were excised after *in vivo* PET imaging on Day 12 and immediately processed for flow cytometry. A single-cell suspension was generated by incubating the tumours in modified RPMI (Gibco) supplemented with 10% heat-inactivated Fetal Bovine Serum (Gibco, Life Technologies), 20 µg/ml DNAse1 (Sigma-Aldrich) and 200 µg/ml Collagenase (Sigma-Aldrich). The samples were mechanically diced and incubated for 1 hour at 37°C and dissociated into single cells by passing through a 100 µm cell strainer. The samples were then counted and assessed for viability with Trypan Blue (Sigma-Aldrich, St. Louis, MO, USA). Cells were stained with antibodies against Kv1.3 (polyclonal FITC; Sigma-Aldrich), CD103 (clone M290 FITC; BD Biosciences, San Jose, CA, USA), CD25 (clone PC61 BB700; BD Biosciences), CD45 (clone 30-F11 BUV395; BD Biosciences), Fixable Live/Dead Blue (Invitrogen, Waltham, MA, USA), CD62L (clone MEL-14 BUV563; BD Biosciences), CD86 (clone GL1 BUV615; BD Biosciences), F4/80 (clone T45-2342; BD Biosciences), NKp46 (clone 29A1.4 BUV737; BD Biosciences), CD3e (clone 500A2 BUV805; BD Biosciences), FoxP3 (clone 150D AlexaFluor647; Biolegend, San Diego, CA, USA), CD44 (clone IM7 APC-R700; BD Biosciences), CD11b (clone M1/70 APC-Cy7; Biolegend, San Diego, CA, USA), Granzyme B (clone QA16A02 PE; Biolegend), CCR7 (clone 4B12 PE-CF594; BD Biosciences), CD19 (clone 6D5 PE-Cy5; Biolegend), CD206 (clone C068C2 PE-Cy7; Biolegend), CD127 (clone SB/199 BV421; BD Biosciences), Ly6G (clone 1A8 BV480; BD Biosciences), CD8 (clone 53-6.7 BV510; BD Biosciences), CD11c (clone N418 BV570; Biolegend), Ly6C (clone HK1.4 BV605; Biolegend), Siglec F (clone E50-2440 BV650, BD Biosciences), CD68 (clone FA-11 BV711; Biolegend), CD4 (clone GK1.5 BV750; BD Biosciences), I-A/I-E (clone M5/114.15.2 BV785; Biolegend).

Tables

Treatment cohort	Days post inoculation	Tumour volume (mm^3 ± SD)
Control	6	137.58 ± 11.84
	9	272.64 ± 15.36
	12	435.45 ± 170.86
	15	682.73 ± 268.37

	21	1473.25 ± 362.07
Treatment Responders (TR) αPD1	6	131.14 ± 11.36
	9	243.69 ± 66.62
	12	203.00 ± 13.64*
	15	236.63 ± 80.47*
	21	598.61 ± 109.20**
Metformin	6	135.57 ± 20.10
	9	285.09 ± 39.05
	12	357.46 ± 84.03
	15	549.53 ± 82.56
	21	1437.41 ± 455.67
αPD1 + Metformin	6	133.94 ± 33.25
	9	244.04 ± 56.97
	12	199.84 ± 115.96*
	15	162.46 ± 132.38**
	21	137.29 ± 123.16***
Treatment Non Responders (TNR)	6	144.60 ± 25.32
	9	272.00 ± 27.19
	12	384.49 ± 113.68
	15	519.30 ± 184.07
	21	1288.59 ± 246.41

Supplementary Table S1. Table showing tumour volumes across each treatment cohort (Control, αPD1, metformin, combined αPD1 + metformin and TNRs). Data are shown as the mean ± S.D; n = 5-10 mice/ group; * $P < 0.05$, ** $P < 0.01$*** $P < 0.001$ compared to TNR.

**Treatment cohort**	**Days post inoculation**	**Tumour volume (mm^3 ± SD)**
Reimplanted TNR	22	102.40 ± 9.22
	32	116.32 ± 39.85
	35	137.91 ± 10.40
	39	190.58 ± 32.80
	42	323.89 ± 185.79
	46	493.09 ± 245.67
Reimplanted αPD1 + Metformin TR	22	105.33 ± 13.05
	32	126.24 ± 12.71
	35	146.88 ± 7.54
	39	165.75 ± 15.33
	42	169.68 ± 19.79
	46	188.78 ± 25.73*

Supplementary Table S2. Table showing tumour volumes in reimplanted αPD1 + Metformin TRs and re-implanted TNRs. Data are shown as mean ± S.D; *n* = 4-6 mice/ group; * $P < 0.05$.

A

	**CD4⁺ % of CD3⁺**	**CD4⁺ Teff% of CD4⁺**	**CD4⁺ T_{CM} % of CD4⁺**	**CD4⁺ Treg % of CD4⁺**
Control	37.10 ± 8.24	73.48 ± 8.64	2.23 ± 1.01	70.50 ± 6.33
TR<br>αPD1	30.01 ± 7.13	70.68 ± 12.66	3.34 ± 1.24	65.01 ± 11.30
Metformin	37.93 ± 7.71	79.75 ± 4.95	1.79 ± 1.09	78.85 ± 5.72
αPD1 + Metformin	32.86 ± 6.92	59.94 ± 15.66	3.67 ± 0.74	64.71 ± 13.23
TNR	32.27 ± 7.46	72.06 ± 9.28	2.89 ± 1.14	73.04 ± 8.91

B

	**CD8⁺ % of CD3⁺**	**CD8⁺ Teff% of CD8⁺**	**CD8⁺ T_{CM} % of CD8⁺**	**CD11b⁺ % of CD45⁺**
Control	50.70 ± 9.83	81.50 ± 8.57	0.49 ± 0.21	14.88 ± 4.81
TR<br>αPD1	62.55 ± 6.60*	78.20 ± 12.64	0.59 ± 0.29	17.43 ± 4.92
Metformin	49.18 ± 9.18	86.11 ± 4.64	0.42 ± 0.21	21.25 ± 2.77
αPD1 + Metformin	69.49 ± 4.89**	76.38 ± 16.10	1.36 ± 0.61*	16.58 ± 5.70
TNR	53.89 ± 5.71	82.10 ± 7.21	0.51 ± 0.29	19.19 ± 3.92

Supplementary Table S3. Table showing the immune cell populations across each treatment cohort (Control, αPD1, metformin, combined αPD1 + metformin and TNRs). A. CD4+, CD4+ T-effector, CD4+ T-central memory and CD4+ T-regulatory cells and B. CD8+, CD8+ T-effector, CD8+ T-central memory and CD11b+ immune cell populations. Data are shown as mean % of cells ± S.D. and are representative of n=5-10 mice/ group, * $P<0.05$; ** $P<0.01$ comparing TR to TNR.

References

1. Tomayko, M.M.; Reynolds, C.P. Determination of subcutaneous tumor size in athymic (nude) mice. *Cancer chemotherapy and pharmacology* **1989**, *24*, 148-154, doi:10.1007/bf00300234.
